# Radiation synergizes with antitumor activity of CD13-targeted tissue factor in a HT1080 xenograft model of human soft tissue sarcoma

**DOI:** 10.1371/journal.pone.0229271

**Published:** 2020-02-21

**Authors:** Caroline Brand, Burkhard Greve, Tobias Bölling, Hans T. Eich, Normann Willich, Saliha Harrach, Heike Hintelmann, Georg Lenz, Rolf M. Mesters, Torsten Kessler, Christoph Schliemann, Wolfgang E. Berdel, Christian Schwöppe

**Affiliations:** 1 Department of Medicine A, Hematology, Oncology, University Hospital Muenster, Muenster, Germany; 2 Department of Radiation Therapy and Radiation-Oncology, University Hospital Muenster, Muenster, Germany; Duke University School of Medicine, UNITED STATES

## Abstract

**Background:**

Truncated tissue factor (tTF) retargeted by NGR-peptides to aminopeptidase N (CD13) in tumor vasculature is effective in experimental tumor therapy. tTF-NGR induces tumor growth inhibition in a variety of human tumor xenografts of different histology. To improve on the therapeutic efficacy we have combined tTF-NGR with radiotherapy.

**Methods:**

Serum-stimulated human umbilical vein endothelial cells (HUVEC) and human HT1080 sarcoma cells were irradiated *in vitro*, and upregulated early-apoptotic phosphatidylserine (PS) on the cell surface was measured by standard flow cytometry. Increase of cellular procoagulant function in relation to irradiation and PS cell surface concentration was measured in a tTF-NGR-dependent Factor X activation assay. *In vivo* experiments with CD-1 athymic mice bearing human HT1080 sarcoma xenotransplants were performed to test the systemic therapeutic effects of tTF-NGR on tumor growth alone or in combination with regional tumor ionizing radiotherapy.

**Results:**

As shown by flow cytometry with HUVEC and HT1080 sarcoma cells *in vitro*, irradiation with 4 and 6 Gy in the process of apoptosis induced upregulation of PS presence on the outer surface of both cell types. Proapoptotic HUVEC and HT1080 cells both showed significantly higher procoagulant efficacy on the basis of equimolar concentrations of tTF-NGR as measured by FX activation. This effect can be reverted by masking of PS with Annexin V. HT1080 human sarcoma xenografted tumors showed shrinkage induced by combined regional radiotherapy and systemic tTF-NGR as compared to growth inhibition achieved by either of the treatment modalities alone.

**Conclusions:**

Irradiation renders tumor and tumor vascular cells procoagulant by PS upregulation on their outer surface and radiotherapy can significantly improve the therapeutic antitumor efficacy of tTF-NGR in the xenograft model used. This synergistic effect will influence design of future clinical combination studies.

## Introduction

Innovative new therapies with defined tumor-associated targets are urgently needed in almost every metastatic malignant disease. New blood vessels invading growing and spreading tumor deposits are essential for oxygen and nutrient supply as well as removal of metabolic waste, and this was reported as one of the hallmarks of cancer [1]. Tumor vasculature exhibits multiple targets for therapeutic approaches such as anti-angiogenic treatment to interfere with formation of neo-vasculature, or vascular disruption and vascular infarction to target existing tumor blood vessels. Denekamp et al. proposed existing tumor vessels and endothelial cells of the tumor vasculature as carrying targets for antivascular treatment approaches [2]. Later, tumor infarction by targeted tissue factor (TF) was proposed [3]. Among multiple potential target molecules on tumor endothelium Pasqualini et al. characterized and proposed small NGR (asparagine-glycine-arginine) containing peptides. These peptides can bind to aminopepdidase N (APN; CD13) [4]. NGR-human tumor necrosis factor (NGR-hTNF), a molecule already studied in clinical trials, was described to particularly bind to a tumor vessel specific CD13 isoform [5]. Additionally, studies with CD13 [6] reported this molecule to play a role in angiogenesis, tumor growth, and also metastasis [7]. However, a prognostic impact of CD13 expression in tumors and tumor vasculature was reported for patients suffering from malignant disease with only some histologies [8–12].

We have designed and produced a large series of fusion proteins with NGR-peptides on the C-terminus of truncated tissue factor (tTF) with the objective of tumor vasculature occlusion by coagulation and resulting infarction [13–17]. A lead protein tTF-NGR (His_tag_-tTF_1-218_-GNGRAHA) has recently entered clinical phase I studies in late stage cancer patients (NCT02902237). tTF-NGR was reported to significantly inhibit growth of human tumor xenografts of different histologies, among them small-cell and non-small cell lung cancer, breast, sarcoma, glioblastoma, and melanoma. As the main mode of action leading to anti-tumor activity of this molecule, tumor vascular thrombosis and infarction has been shown by different *in vivo* or *ex vivo* imaging methods [14–17].

Since monotherapy with tTF-NGR only lead to tumor growth inhibition and only rarely to complete tumor remissions, and since we have observed combinatorial therapeutic effects of tTF-NGR combined with cytotoxic anticancer drugs such as doxorubicin when given in specific pharmacokinetically defined sequences [18], we here have investigated possible synergistic effects of tTF-NGR with radiotherapy. Synergistic therapeutic effects by combining these two treatment modalities were observed in a human sarcoma xenograft model and mechanistic studies suggest radiation-induced increased phosphatidylserine (PS) concentration in the tumor vasculature leading to higher procoagulant effects of tTF-NGR as a molecular basis.

## Materials and methods

### Cell lines

Handling of cell lines was described previously [18]. In brief, human umbilical vein endothelial cells (HUVEC; PromoCell, Heidelberg, Germany) were exclusively used at low passage numbers. Cells were cultured in MCDB 131 medium supplemented with 20% fetal calf serum (FCS), 2 mM glutamine (Gibco, Eggenstein, Germany), 50 μg/ml endothelial cell growth supplement (ECGS; Sigma, Taufkirchen, Germany), 5 U/ml heparin (Sigma, Taufkirchen, Germany), and kept at 37°C in 5% CO_2_ and high humidity. Cell culture dishes were coated with 0.2% gelatine. The human HT1080 fibrosarcoma cell line was purchased from ATCC (Manassas, VA, USA; RRID: CVCL_0317) and cultured in Dulbecco´s medium (Gibco) supplemented with 10% FCS. Cell line identity was regularly authenticated and confirmed by short tandem repeat (STR) profiling. Both cells stained positive for CD13, the binding target of tTF-NGR, with or without irradiation.

### tTF-NGR

Good Manufacturing Practice (GMP) cloning, expression and purification of His_tag_-tTF_1-218_-GNGRAHA has been described in detail before [14,15]. Experiments were mainly done with clinical grade tTF-NGR material.

### Irradiation of cells in vitro

HUVEC (6 x 10^5^ cells per flask) and HT1080 (2 x 10^5^ cells per flask) cells were seeded into T25 culture flasks of identical size and preincubated for 18 h to guarantee identical confluency at the time of irradiation (approx. 80% confluency). Cells were then irradiated *in vitro* using a TrueBeam linear accelerator (Varian, Palo Alto, CA, USA). Dosimetry was done by the dose-control system within the accelerator with an automatic switch-off mechanism. Doses of 4 and 6 Gy were applied at a dose rate of 4.8 Gy per minute. After screening a broader dose range, we have chosen these lower doses, since the relation of cells in early apoptosis (PS-positive and propidium iodide (PI)-negative, see below) versus destroyed cells (PS and PI-positive) was advantageous for performing the Factor X assay with identical numbers of PS-positive but otherwise intact (PI-negative) cells (see below). After irradiation cells were cultivated at 37°C and 5% CO_2_ for further 24 h, 48 h or 72 h, respectively, and then analyzed for their surface phosphatidylserine (PS) concentration by flow cytometry.

### Annexin V and propidium iodide staining and flow cytometric analysis

General methods have been decribed before [18]. Briefly, Annexin V staining of phosphatidylserine (PS) in the outer leaflet of the phospholipid bilayer of a cellular membrane using flow cytometry is widely used as a standard assay for cellular apoptosis, as increase of PS staining is observed to be directly connected with early apoptosis. PS staining technique was described in detail earlier [18] and applied with minor modifications: After a post-incubation time of 24 h, 48 h or 72 h, respectively, irradiated cells and untreated control cells were harvested, washed in phosphate-buffered saline (PBS) and each 1x10^5^ cells were resuspended in 500 μl binding buffer. To measure the surface concentration of PS on early apoptotic cells, samples were stained with Annexin V fluorescein isothiocyanate (FITC) at a final concentration of 0.375 μg/ml (Becton Dickinson, San Jose, CA, USA) according to the manufacturer’s instructions. To distinguish between early apoptotic cells with intact cellular membranes and necrotic or late-apoptotic cells with cellular membranes destroyed and thus permeable for intracellular material such as nucleic acids, 1 μg of the nucleic acid binding propidium iodide (PI) was added to each sample. The cells were subsequently incubated for 10 minutes at room temperature in the dark. For cytometric analysis we used a FACSCalibur flow cytometer (Becton Dickinson), and cells were washed twice and finally resuspended in 500 μl binding buffer before analysis. For each measurement, 1x10^4^ cells were counted and results were analyzed with the CellQuest Pro software (Becton Dickinson). Experiments were done at least 3 times.

### Factor X activation assay

Factor X (FX) activation assay is a validated quality control assay in our Good Manufacturing Practice (GMP)-laboratory and was described earlier [18]. The assay was originally established and reported by Ruf et al. [19] and is based on the ability of tTF-NGR to enhance the specific proteolytic activation of FX by a complex of tissue factor (TF), FVIIa, and FX (tTF-NGR:FVIIa:FX instead of TF:FVIIa:FX). In brief, 20 μl of the following was added to each well in a microtiter plate: (a) 50 nM recombinant FVIIa (Novo-Nordisc, Bagsværd, Denmark) in Tris-buffered saline (TBS) containing 0.1% bovine serum albumin (BSA); (b) 750 pM tTF-NGR in TBS-BSA; (c) 25 mM CaCl_2_, and in place of phospholipids (d) 5,000 or 10,000 non-irradiated or irradiated HUVEC or HT1080 cells with a post-incubation time of 48 h at 37°C and 5% CO_2_ or control cells. Radiation doses (4 and 6 Gy) were also chosen to upregulate PS, but leave the bulk of cells undestroyed (PI negative or low), to be able to add exactly counted and thus identical cell numbers to the Factor X assay. Constant tTF-NGR concentration was chosen on the basis of the K_D_ values in our validated Factor X assay used for quality control of the clinical tTF-NGR substance. To mask and inhibit PS on HUVEC or HT1080 cell surfaces, cells were incubated with 5 (+) or 10 (++) μg/10^5^ cells of recombinant human Annexin V (Becton Dickinson, San Jose, CA, USA) for 15 minutes before any reagents were added. After 10 minutes at room temperature, the substrate FX (Enzyme Research Laboratories, Swansea, UK) was added (final concentration 1 μM). After further 10 minutes, the reaction was stopped in 100 mM EDTA; and Spectrozyme FXa (American Diagnostica, Greenwich, USA; final concentration 0.7 mM) was added immediately prior to analysis on a microplate reader (Bio-Rad, München, Germany). Pro-coagulant activity within the assays without irradiation of the HUVEC or HT1080 cells was set as 100%.

### Xenograft model

Basic experimental procedures have been recently published by us [18]. In brief, CD-1 nude mice were purchased from Charles River Laboratories (Sulzfeld, Germany) and acclimated to our animal-experiment facility for at least 1 week before any experimentation. Mice were kept in individually-ventilated cages (IVC) on a 12:12 h light:dark cycle in a low-stress environment (22°C, 50% humidity, low noise) and given food and water *ad libitum*. All procedures on animals were performed in agreement with German regulations (Tierversuchsgesetz §8, Abs. 2) and approved by the North-Rhine Westfalian “Landesamt fuer Natur, Umwelt und Verbraucherschutz NRW” (LANUV) in form of a specific project license (50.0835.1.0 (G35/2005)). Single HT1080 cell suspensions (5 x 10^6^ per mouse) were injected subcutaneously (s.c.) into the flank of female CD-1 nude mice. Tumor growth was observed to a mean volume as indicated in the Results section. Subsequently, mice were randomly assigned to different experimental groups. Tumors were irradiated with 6 MV Photons from a linear accelerator (Clinac, Varian Medical Systems, Palo Alto, USA) with a dose rate of 4.8 Gy/min at room temperature while shielding of the remaining mouse body. The mice were fixed in special boxes with anesthesia by an intraperitoneal injection of ketamine (125 mg/kg body weight [b.w.]) and xylazine (12.5 mg/kg b.w.) according to the animal project licence. A tissue equivalent material was used to reduce the build-up effect. The dose distribution was verified using thermoluminescence dosimetry. tTF-NGR or control 0.9% NaCl solution (saline control) were slowly applied intravenously (i.v.) via the tail veins in the indicated doses and time schedules. Tumor size was evaluated using a standard caliper measuring tumor length and width; tumor volumes were calculated using standardized formulas [18]. According to our project license, animals had to be sacrificed when tumors became too large, if mice lost too much body weight, or at signs of pain. In this case, mice were sacrificed by cervical dislocation in deep ketamine/xylazine anesthesia in agreement with standard regulations and the project license.

### Statistical analysis

Statistical significance of differences were tested by *t*-test or by Mann-Whitney rank sum test for independent groups. For the *in vitro* experiments we have used the *t-*test (2-tailed). The Mann-Whitney test was used for the tumor growth comparison in the tumor therapy experiments. Since we have a large Gaussian normal value distribution, we have used the *t-*test for the mouse body weight comparisons. Two-tailed P values </ = 0.05 were considered as indicating significant differences. All data are presented in means with standard error (SE) bars.

## Results

### In vitro irradiation increases phosphatidylserine (PS) concentration on the outer cell surface of human endothelial and HT1080 sarcoma cells

**Fig 1:**
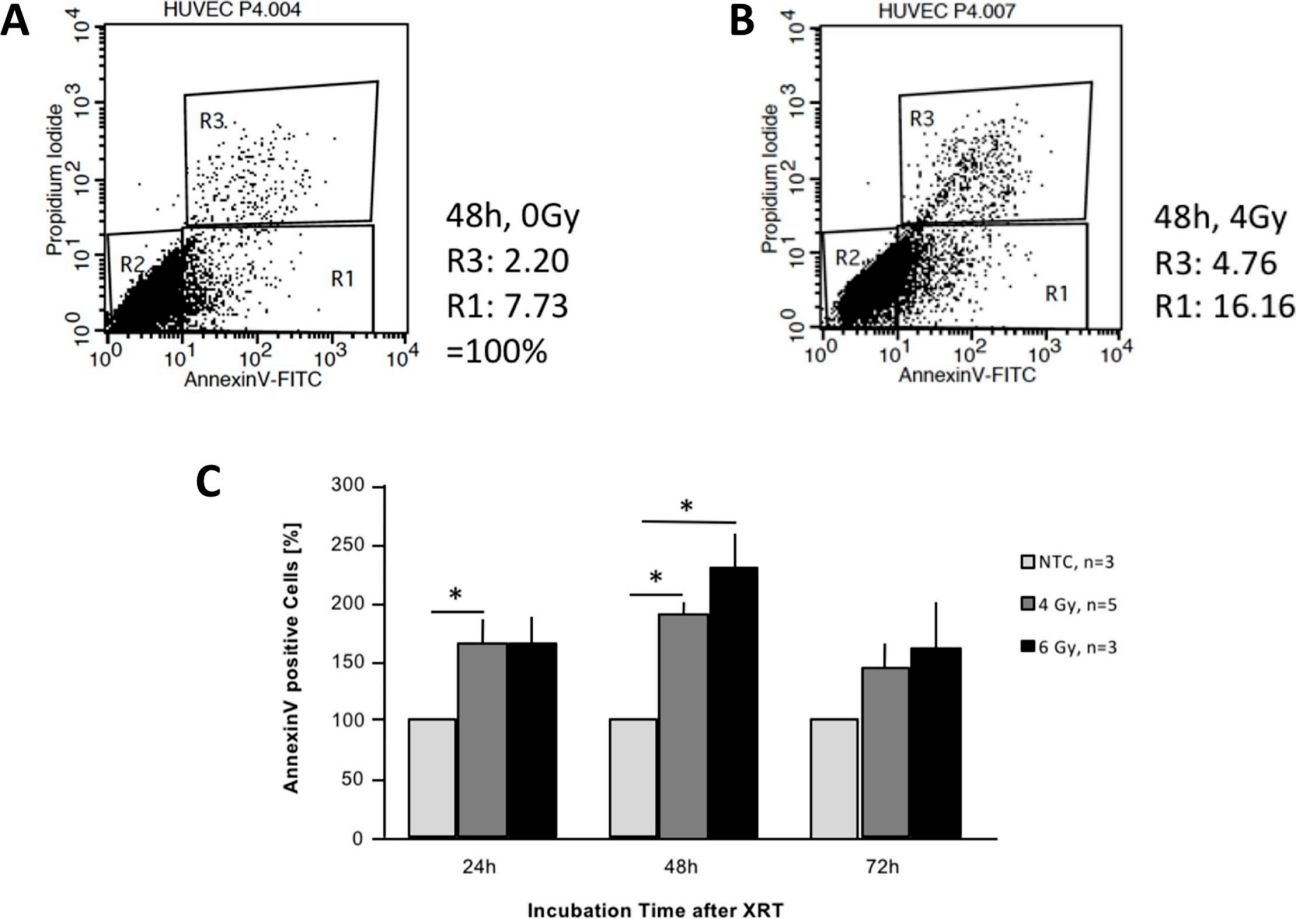
Upregulation of phosphatidylserine (PS) on the cell surface of human endothelial cells (HUVEC) by radiation *in vitro*. (A) PS (Annexin V positive, R1) versus propidium iodide (PI; R3) staining without irradiation (control) after 48 hours of incubation. 2.2% of the cells were PI-positive (necrotic) and 7.73% of the cells stained positive for PS. (B) PS versus PI staining after irradiation with 4 Gy and 48 hours of incubation. (C) Quantitative results of all independent experiments (3- to 5-fold for control and 6 Gy, 5-fold for 4 Gy). Asterisks represent comparisons with two-sided p-values <0.05 (24 hours: control vs. 4 Gy, p = 0.022; control vs. 6 Gy, p = 0.092 (n was only 3, variation was larger). 48 hours: control vs. 4 Gy, p = 0.0002; control vs. 6 Gy, p = 0.043). Values at 72 hours were not significant since spontaneous apoptosis of the cells without radiation varied and increased. The no-treatment control (NTC) values have been normalized to 100% and the other values have been given as mean percentages of control with standard error (SE) bars.

We have irradiated HUVEC and HT1080 sarcoma cells, seeded at 6 x 10^5^ (HUVEC) and 2 x 10^5^ (HT1080) cells into culture flasks and preincubated for 18 h, *in vitro* with different dosing (0, 4, 6, and 8 Gy) and time (24, 48, 72 h incubation after irradiation) schedules to induce apoptosis with PS externalization into the outer cellular membrane. Following irradiation and incubation, we have subjected the cells to flow cytometry for phosphatidylserine (PS) and propidium iodide (PI). After screening this wider range of experimental conditions, we have standardized experiments to 4 and 6 Gy, as we here have observed satisfactory upregulation of PS and still tolerable values of cellular destruction (PI). As was shown by flow cytometry before and after irradiation, PS concentrations on the cell surface of HUVEC (Fig 1) could be significantly increased by irradiation as a sign for early apoptosis, whereas cells were not completely destroyed (PI-positive) so that they could be further used at identical cell numbers for FX assays. Fig 1A and 1B show one representative experiment without (A) and with 4 Gy (B) irradiation out of 3 independent experiments. Fig 1C shows the complete sets of different radiation doses with the numbers of independent experiments given as n (n number variation due to technical reasons). As can be seen PS was e.g. upregulated approx. 2.5 times under 6 Gy at 48 h, with >95% of the cells not being necrotic (Fig 1C). Values at 72 hours were lower and differences not significant since spontaneous apoptosis of the cells varied more without radiation and PI staining increased, which was deduced from the total to get the numbers of exclusively PS-positive cells being of interest here.

**Fig 2:**
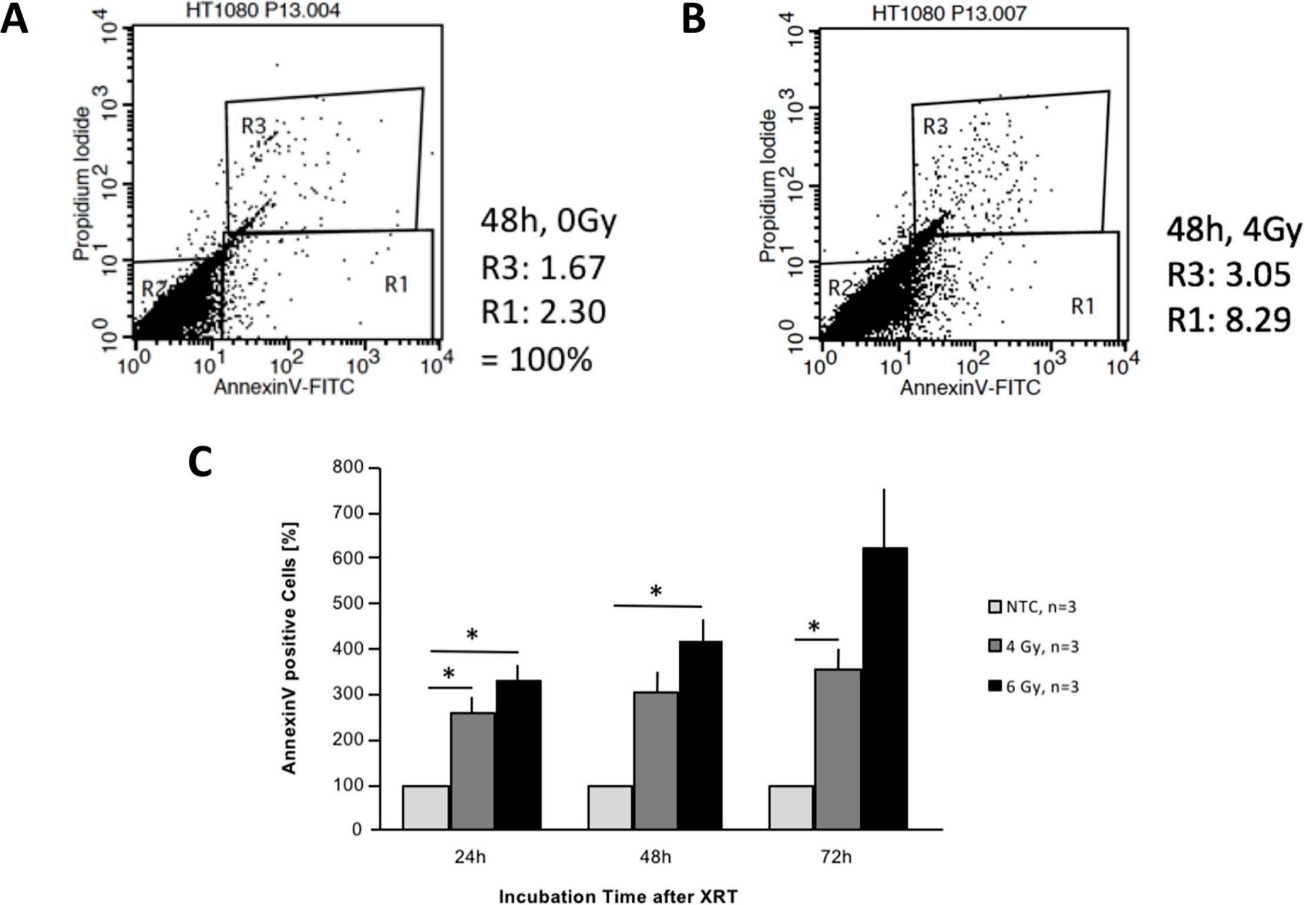
Upregulation of phosphatidylserine (PS) on the cell surface of human sarcoma cells (HT1080) by radiation *in vitro*. (A) PS (Annexin V positive, R1) versus propidium iodide (PI; R3) staining without radiation (control) after 48 hours of incubation. 1.67% of the cells were PI-positive (necrotic) and 2.30% of the cells stained positive for PS. (B) PS versus PI staining after radiation with 4 Gy and 48 hours of incubation. (C) Quantitative results of all independent experiments (n). Asterisks represent comparisons with two-sided p-values <0.05 (24 hours: control vs. 4 Gy, p = 0.047; control vs. 6 Gy, p = 0.027. 48 hours: control vs. 4 Gy, p = 0.05; control vs. 6 Gy, p = 0.019. 72 hours: control vs. 4 Gy, p = 0.037; control vs. 6 Gy p = 0.06). The no-treatment control (NTC) values have been normalized to 100% and the other values have been given as mean percentages of control with standard error (SE) bars.

Next, we tested irradiation effects on the human sarcoma cell line HT1080, since also tumor cells can express CD13 and form part of the inner cell lining of the tumor neo-vasculature (see below), and tTF-NGR might be pro-coagulatory also within these regions. Similar results were obtained irradiating and testing these cells. HT1080 was considerably less sensitive to *in vitro* cultivation but more sensitive to radiation damage than the endothelial cells. Fig 2A and 2B show one representative experiment out of 3 independent experiments without (A) and with 4 Gy (B) irradiation. Fig 2C shows the complete sets of different radiation doses with the numbers of independent experiments given as n. Spontaneous signs of early apoptosis were lower, but irradiation with e.g. 6 Gy and a following incubation for 48 h upregulated PS expression approx. 4-fold, with >95% of the cells not being necrotic (Fig 2C).

Taken together, we have shown significant upregulation of PS as an early sign of apoptosis by irradiation with otherwise intact cells, which could be used in following Factor X assays.

### Increased pro-coagulant activity of irradiated human endothelial and sarcoma cells and dependence from phosphatidylserine (PS) on the outer cell surface

**Fig 3:**
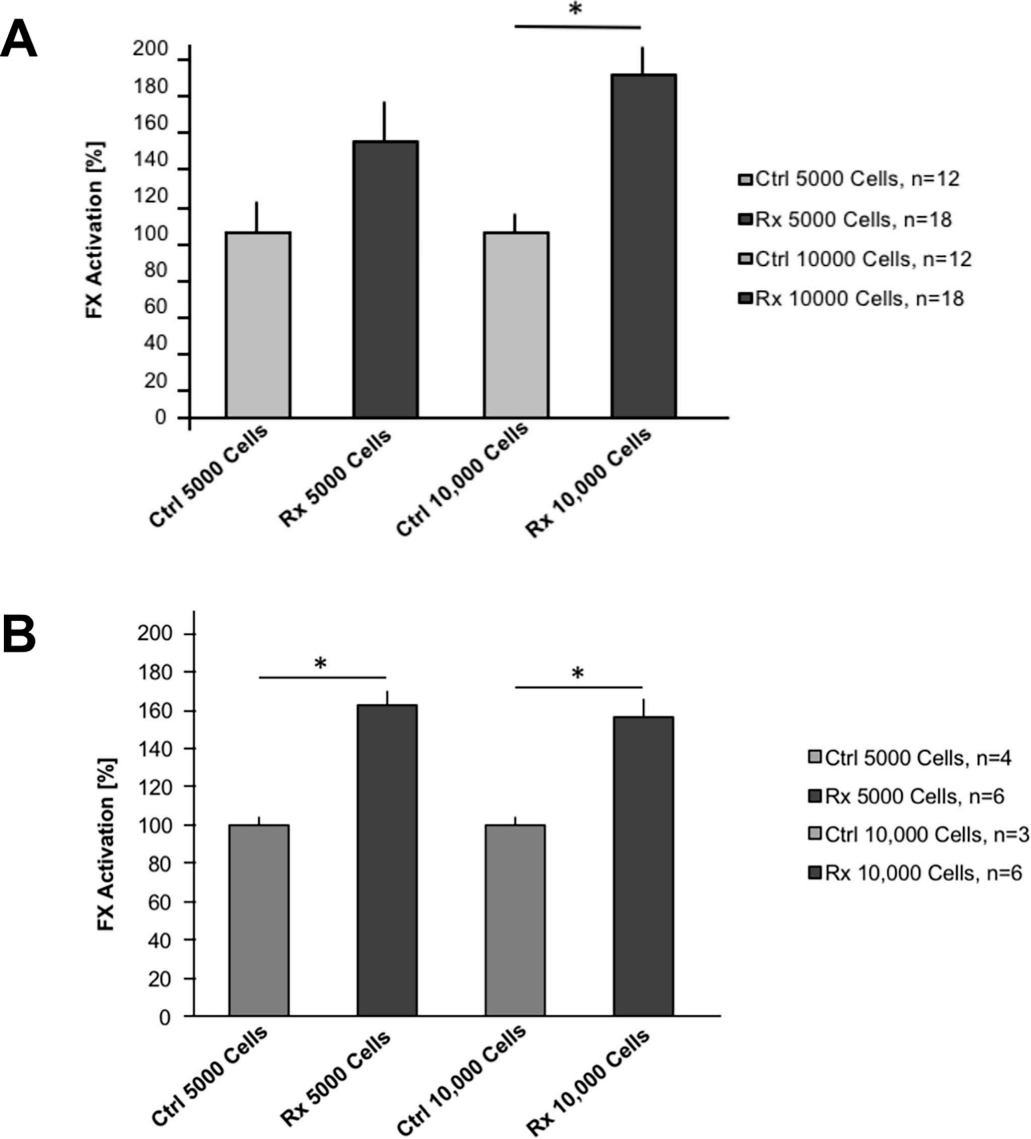
Factor X activation assay in the presence of human endothelial cells or human sarcoma cells. (A) Different numbers of human endothelial cells (HUVEC; 5000 or 10,000 cells per assay) were tested without or with previous irradiation in a validated FX activation assay as described in the Methods section with identical amounts of tTF-NGR and all other test components present. Results with cells without previous irradiation (Ctrl, control) were given as 100%. Results with cells after irradiation (Rx, 4 Gy) are represented by the black columns. Results of 3 independent experiments with total numbers of assays given as n are shown with mean values and standard error (SE) bars. Asterisk represents comparisons with two-tailed p values <0.05 (5000 cells: control vs. 4 Gy, p = 0.074; 10,000 cells: control vs. 4 Gy, p = 0.0001). (B) Different numbers of human sarcoma cells (HT1080) were tested in the FX activation assay as described for (A). Results of FX experiment with total numbers of assays given as n are shown with mean values and standard error (SE) bars. Asterisks represent comparisons with two-tailed p values <0.05 (5000 cells: control vs. 4 Gy, p = 0.0001; 10,000 cells: control vs. 4 Gy, p = 0.00461).

Experiments by different laboratories have shown phospholipid/PS-dependency of the pro-coagulant state [20–22]. Thus, we hypothesized that increasing PS externalization to the outer cell surface of endothelial and tumor cells in the process of apoptosis induced by radiation can lead to a better pro-coagulant milieu and consecutively to a more efficient factor X (FX) activation at equimolar concentrations of tTF-NGR present in the membrane bound tTF-NGR:FVIIa:FX complex resulting in FX activation. In the next set of 3 independent experiments shown in Fig 3A (n, total numbers of assays of all experiments) we have subjected HUVEC after irradiation with 4 Gy and a post-incubation time of 48 h to our FX activation assay. The assay was performed with equal amounts of tTF-NGR present together with the cells. We have performed the FX assay with two different numbers of cells in the assay (5000 or 10,000 cells) to rule out a cell number effect.

Indeed, as tested by this comparative FX activation HUVEC with equimolar concentrations of tTF-NGR and other components in the assay revealed a significantly higher procoagulant state after irradiation than without irradiation (Fig 3A). In the experiment shown in Fig 3B the same effect could be shown with HT1080 cells (Fig 3B).

**Fig 4:**
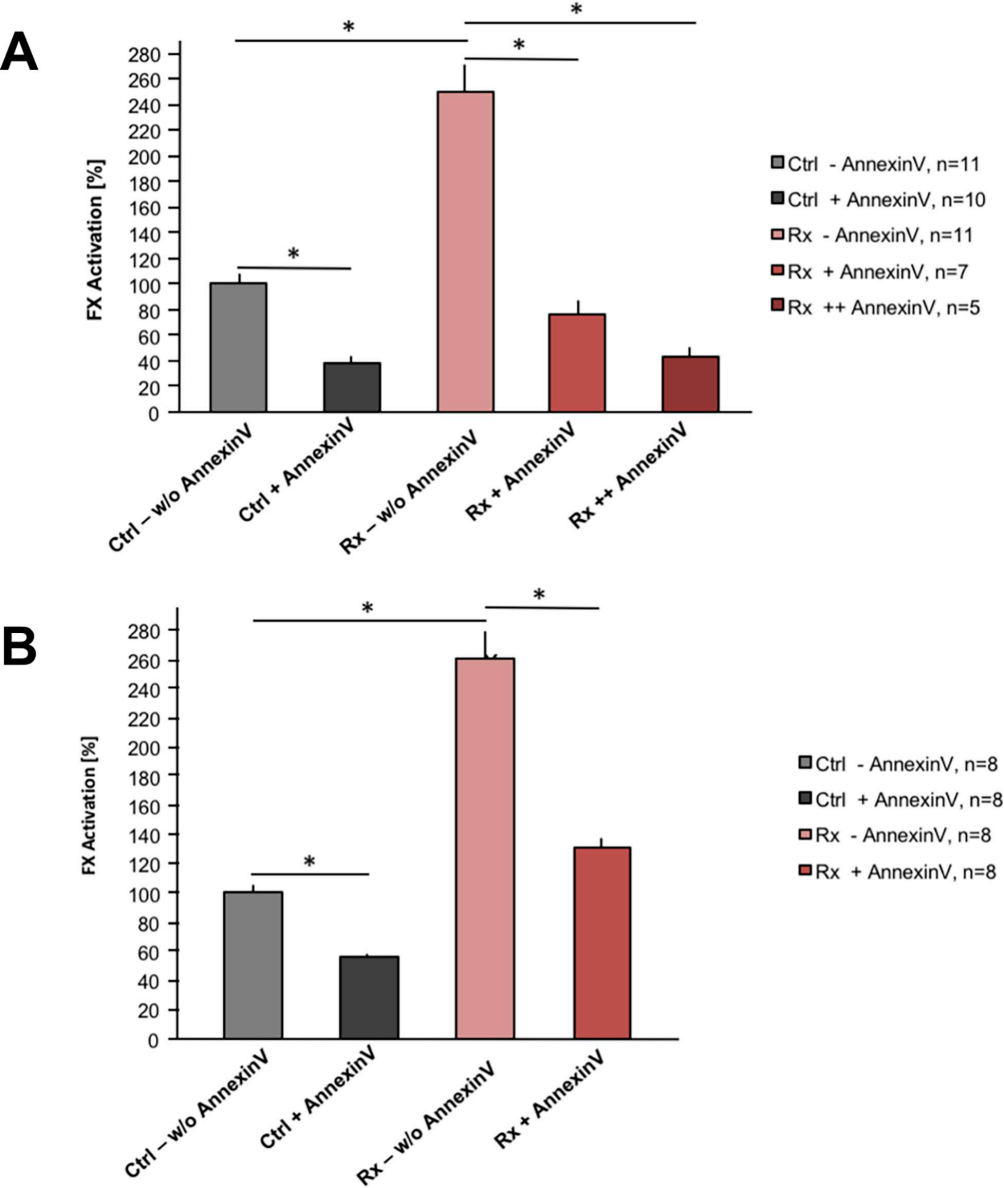
Factor X activation assay in the presence of human endothelial cells or human sarcoma cells with the variables of preceding irradiation and Annexin V incubation. (A) Human endothelial cells (HUVEC) were tested without (grey columns) versus with (red columns) previous irradiation (4 Gy) in a validated FX activation assay as described in the Methods section with identical amounts of tTF-NGR and all other test components present. Tests with 5 (+) or 10 (++) μg/10^5^ cells of recombinant human Annexin V versus without (w/o) previous incubation of the cells with Annexin V (binding and masking PS) were compared. Results with cells without previous irradiation and without Annexin V incubation (Ctrl, control; w/o, without Annexin V) were given as 100%. Results with cells after irradiation (Rx, 4 Gy) are represented by the red columns (light red, without and dark red, with previous incubation with Annexin V). Results of 3 different experiments with numbers of assays given as n are shown with mean values and standard error (SE) bars. Asterisks represent comparisons with two-tailed p values <0.0001. (B) Human sarcoma cells (HT1080) were tested in the FX activation assay as described for (A). Results of 2 different experiments with numbers of assays given as n are shown with mean values and standard error (SE) bars. Asterisks represent comparisons with two-tailed p values <0.0001.

To further proof causal relationship between higher pro-coagulant state of the cells and PS externalization following irradiation, we masked PS on the cells by pre-incubation with Annexin V before subjecting them to the FX activation assay. In 3 independent experiments we could show, that irradiated HUVEC (Fig 4A; n, total numbers of assays of all experiments) lost pro-coagulant properties upon pre-incubation with and PS masking by Annexin V. For the HUVEC experiment, we tested a dose range of Annexin V in addition and observed a dose-effect relationship (Fig 4A). As the proposed mechanism possibly is also relevant for the combinatorial effects of radiation and tTF-NGR *in vivo* when tumor cells are forming smaller areas of the inner cell layer of the tumor neovasculature, we have repeated the experiment with HT1080 cells testing one Annexin V concentration, and observed an identical “masking effect” (Fig 4B; 2 independent experiments, n represents total numbers of assays of all experiments). Interestingly, in both cell types the basis presence of PS in the outer layer of the cell surface membrane without irradiation lead to a certain pro-coagulant activity which could be significantly reduced by masking PS with Annexin V (Fig 4A and 4B, grey and dark grey columns).

### Combination of radiotherapy with tTF-NGR in vivo using the HT1080 xenograft model

**Fig 5:**
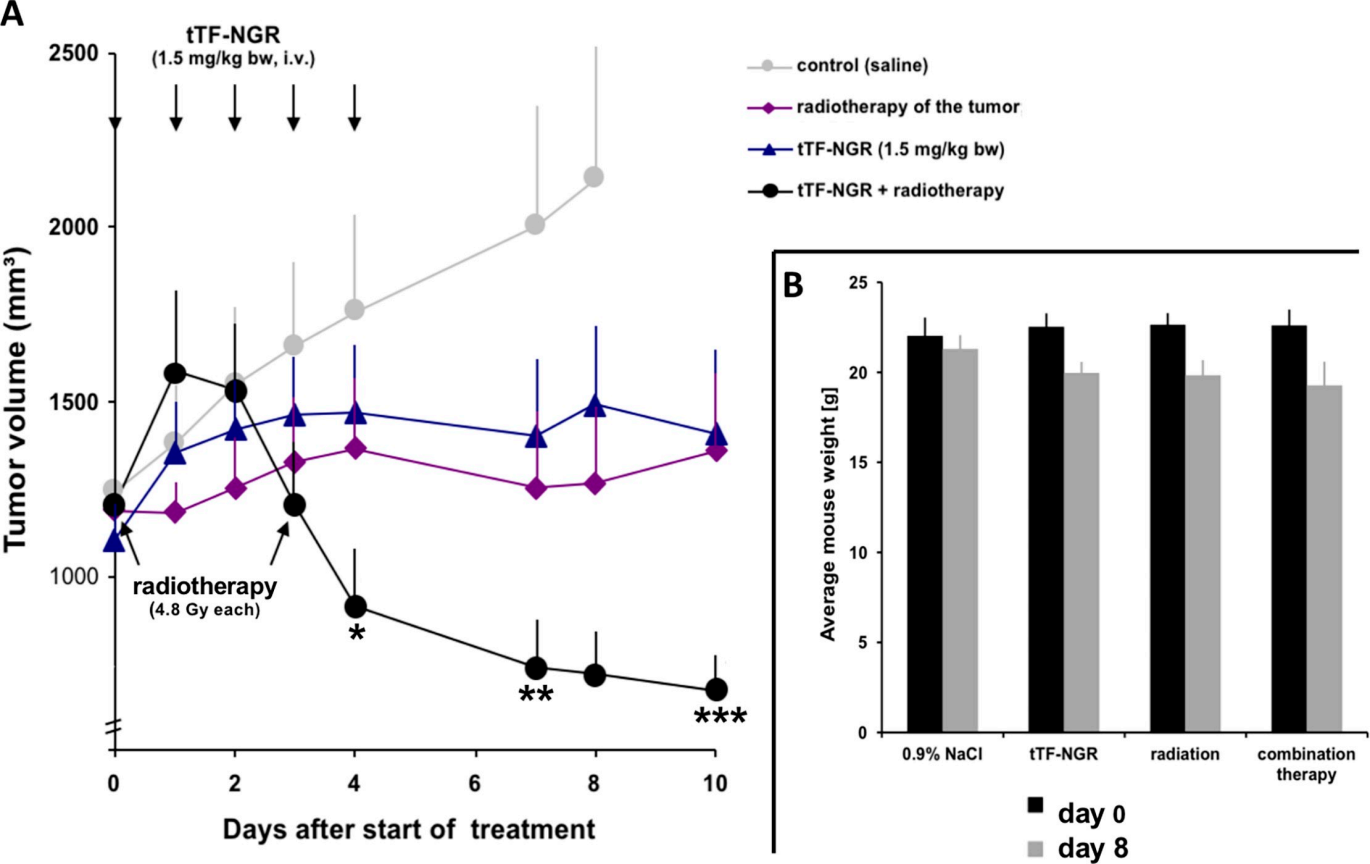
HT1080 human sarcoma xenograft growth in CD-1 athymic mice and body weight comparison under therapy with intravenous (i.v.) tTF-NGR, tumor radiotherapy, and a combination of both therapeutic modalities, respectively. (A) Arrows show treatment intervention for tTF-NGR (1.5 mg/kg body weight (bw) x 5) or tumor radiotherapy (4.8 Gy x 2). Control group (0.9% NaCl (= saline) i.v. at identical days as tTF-NGR, n = 6) is represented by grey dots, tTF-NGR group (n = 5) by blue triangles, radiotherapy (n = 8) by red diamonds, and combination (n = 6) by black dots. Given are mean values plus standard error (SE) bars. *, first day of significant tumor size difference between combination therapy and control (Mann-Whitney rank sum test, two sided p value <0.05); **, significant tumor size difference between tTF-NGR monotherapy and combination (two sided p value <0.05); ***, significant tumor size difference between irradiation monotherapy and combination therapy (two sided p value <0.05). (B) Body weight (b.w.) comparison of the mice at the beginning and at the end of the observation (day 8). Given are mean values (g, gram) with standard error (SE) bars. Dark grey columns represent b.w. before start of experiment, light grey columns represent b.w. at the end of observation (day 8). Statistical comparison (*t-*test) revealed b.w. decrease, when values before treatment and at day 8 were compared: two-tailed p-values for 0.9% NaCl control (p = 0.1449), tTF-NGR (p = 0.0211), radiation (p = 0.0006), combination treatment (p = 0.015), with significant decrease for all groups except the controls. When the b.w. values at day 8 were compared between the different groups there were no significant differences (all two-tailed p-values > 0.2) indicating no significantly lower b.w. in any group as compared to any other.

Since we were able to show that cells forming the tumor vasculature, such as endothelial cells and tumor cells, can be rendered pro-coagulant by radiation-induced externalization of PS and thereby can increase the pro-coagulant activity of tTF-NGR, we have tested combination therapy with tTF-NGR and regional tumor irradiation in a human sarcoma xenograft system *in vivo*. Combination of regional tumor radiotherapy with systemic tTF-NGR showed significantly higher therapeutic activity against HT1080 fibrosarcoma xenografts than either of the treatment modalities alone. Whereas both therapeutic modalities at the doses applied alone effectively stopped tumor growth, combination therapy lead to tumor shrinkage with better outcome at the end of the experiment. Fig 5A depicts one of two experiments, with the second showing additive effects.

Tolerability of the treatment in general was good. There were modest, but significant body weight reductions in all treatment groups during therapy except in the saline group (body weight reduction non-significant), however, with no significant differences of body weight between the groups at the end of observation (Fig 5B). Body weight differences have to be interpreted with due caution, since the influence by the tumor-disease and the differences in tumor growth and weight between the groups might interfere. Further, some of the animals injected with tTF-NGR developed tail vein thrombosis and necrosis at the injection site of the tail, probably due to the high concentrations of the substance injected in a short time with vascular damage by the injection needle. Otherwise, observation revealed no toxicity of the different treatments.

## Discussion

Earlier reports by different laboratories have described phospholipid/PS-dependency of the pro-coagulant state [20–22]. Furthermore, an increasing PS externalization to the outer leaflet of the cellular phospholipid bilayer has been shown to be achieved by pro-apoptotic maneuvers [22–24]. In consequence of both, i.e. dependence of pro-coagulant state by PS presence on outer cell surface and induction of this state by apoptotic stimuli, we and other groups could show increased pro-coagulant activity of endothelial cells by PS externalization induced by cytotoxics such as doxorubicin [18, 25, 26] or by low-energy ultrasound [27].

In this study, we have observed significantly improved antitumor therapeutic activity of a combination using regional ionizing tumor irradiation with systemic tTF-NGR. Our results further suggest that this synergism is based on the radiation-induced early-apoptotic externalization of PS on stimulated HUVEC, which increases the pro-coagulant activity of tTF-NGR as the important mechanism of action of this molecule leading to tumor vascular thrombosis, infarction, and tumor growth inhibition. Since tumor cells, such as HT1080 sarcoma cells, express CD13 and can also form part of the inner cellular lining of tumor neo-vasculature, this being described as vascular mimicry [28], we have also tested the pro-coagulant behaviour of HT1080 sarcoma cells following irradiation as a further mechanism explaining the combinatorial effect, and have obtained similar results as with stimulated HUVEC. In this respect it is of interest, that the anti-PS antibody Bavituximab, which was tested in clinical trials with cancer patients (NCT01999673), can be enhanced in its antitumor efficacy by radiation [29]. The dose for *in vivo* irradiation in the experiments reported here was chosen to be therapeutically active against the tumor, but still tolerable for the animals according to a previous experimental series [30]. Spot checks for macroscopic and microscopic organ damage in animals after stop of the experiments revealed no specific treatment effects.

The authors can envisage the following limitations of this study. HUVEC taken as model cell system in our study, although serum-stimulated, are biologically not exactly identical to tumor neovasculature endothelial cells, and further, the hypothesis that radiation-induced apoptotic PS upregulation and subsequent higher pro-coagulant state of cells forming the inner layer of the tumor neovasculature is shown to be operative *in vitro*, but formal *in vivo* proof is not presented. However, we have seen comparable effects with another regional treatment, low-energy ultrasound treatment [27] and other confounding factors such as intratumoral entrapment of cytotoxics, as observed with doxorubicin combinations [18], should not interfere in this study. Further, we cannot exclude regrowth of the treated tumors after stop of treatment, since animal protection regulations require to stop the complete experiment when tumor size has reached a certain size in the first group. Finally, we have restricted our therapeutic experiments to one xenograft model. However, since xenografts from tumors of different germ layers when transplanted as single cell suspensions are supported by a tumor vascular system originating from the host, and since CD13 on the tumor vascular cells is the main target for tTF-NGR, this synergistic effect may be also operative in models using other tumor histologies.

Our experiments reported here are part of the examination of a larger series of fusion proteins targeting truncated tissue factor (tTF) to the tumor vasculature by small NGR-peptides at the C-terminus of tTF with the result of tumor vessel thrombotic occlusion, infarction, and human tumor xenograft growth inhibition [13–18]. The lead protein, tTF-NGR (His_tag_-tTF_1-218_-GNGRAHA), has recently entered a clinical phase I dose-escalation study in patients with advanced solid tumors or lymphomas beyond standard therapies (NCT02902237). Its main binding target is the aminopeptidase N (APN; CD13) [6], a transmembrane enzyme expressed on a variety of tissues and cells, which is upregulated on endothelial cells in tissues with active formation of new blood vessels, such as in tumors of various histologies, and which has been described as being operative for capillary tube formation [6, 31]. Thus, CD13 is often reported as a target in studies describing experimental imaging of tumors by a variety of molecules and methods [32–34]. Interestingly, NGR-coupled to the N-terminus of TNF has been reported to bind specifically to a CD13 isoform preferentially present in the tumor vascular cells [5]. However, this was found with an NGR-coupled TNF having other amino-acid flanking regions than our tTF-NGR protein.

Treating HT1080 and other human tumor xenografts with tTF-NGR alone or in combination, intratumoral blood pooling and vascular disruption by tumor vascular occlusion through thrombosis was described as the main mode of action of this new therapeutic approach [13–18]. It was possible to directly visualize intravascular fibrin deposition as the consequence of tTF-NGR application by fluorescence reflectance imaging with fluorochrome-labelled fibrinogen [15, 17].

At present, two NGR-peptide linked investigational medicinal products are in clinical trials in cancer patients. Besides tTF-NGR, being in clinical phase I (NCT02902237), NGR-human tumor necrosis factor (NGR-hTNF) is studied in phase II and phase III [35–37], and recent phase III results (NCT01098266) are published. Further, CD13 is targeted by a variety of different compounds other than NGR-molecules [38]. As an example, in a randomized trial postoperatively given bestatin has been reported to improve overall survival of patients with stage I squamous-cell lung cancer [39]. This further supports the possible role of CD13 as a tumor vascular target for new cancer therapies.

## Conclusion

In conclusion, radiation therapy and systemic tTF-NGR have synergistic antitumor efficacy. As similar effects can also be observed with combinations of cytotoxics and tTF-NGR, further clinical trials should focus on combination protocols.

## References

[1] Hanahan D, Weinberg RA. Hallmarks of cancer: the next generation. Cell 2011;144:646–74. 10.1016/j.cell.2011.02.013 21376230

[2] Denekamp J: Endothelial cell proliferation as a novel approach to targeting tumour therapy. Br J Cancer 1982;45:136–39. 10.1038/bjc.1982.16 7059456 PMC2010961

[3] Huang X, Molema G, King S, Watkins L, Edgington TS, Thorpe PE: Tumor infarction in mice by antibody-directed targeting of tissue factor to tumor vasculature. Science 1997;275:547–50. 10.1126/science.275.5299.547 8999802

[4] Pasqualini R, Koivunen E, Kain R, Lahdenranta J, Sakamoto M, Stryhn A, et al: Aminopeptidase N is a receptor for tumor-homing peptides and a target for inhibiting angiogenesis. Cancer Res 2000;60:722–27. 10676659 PMC4469333

[5] Curnis F, Arrigoni G, Sacchi A, Fischetti L, Arap W, Pasqualini R, et al: Differential binding of drugs containing the NGR motif to CD13 isoforms in tumor vessels, epithelia, and myeloid cells. Cancer Res 2002;62:867–74. 11830545

[6] Wickström M, Larsson R, Nygren P, Gullbo J: Aminopeptidase N (CD13) as a target for cancer chemotherapy. Cancer Science 2011;102:501–08. 10.1111/j.1349-7006.2010.01826.x 21205077 PMC7188354

[7] Guzman-Rojas L, Rangel R, Salameh A, Edwards JK, Dondossola E, Kim YG, et al: Cooperative effects of aminopeptidase N (CD13) expressed by nonmalignant and cancer cells within the tumor microenvironment. Proc Natl Acad Sci USA 2012;109:1637–42. 10.1073/pnas.1120790109 22307623 PMC3277167

[8] Tokuhara T, Hattori N, Ishida H, Hirai T, Higashiyama M, Kodama K, et al: Clinical significance of aminopeptidase N in non-small cell lung cancer. Clin Cancer Res 2006;12:3971–78. 10.1158/1078-0432.CCR-06-0338 16818694

[9] Ikeda N, Nakajima Y, Tokuhara T, Hattori N, Sho M, Kanehiro H, et al: Clinical significance of aminopeptidase N/CD13 expression in human pancreatic carcinoma. Clin Cancer Res 2003;9:1503–08. 12684426

[10] Hashida H, Takabayashi A, Kanai M, Adachi M, Kondo K, Kohno N, et al: Aminopeptidase N is involved in cell motility and angiogenesis: its clinical significance in human colon cancer. Gastroenterology 2002;122:376–86. 10.1053/gast.2002.31095 11832452

[11] Schmidt LH, Brand C, Stucke-Ring J, Schliemann C, Kessler T, Harrach S, et al: Potential therapeutic impact of CD13 expression in non-small cell lung cancer. PLoS One 2017;12:e0177146 10.1371/journal.pone.0177146 28604784 PMC5467809

[12] Surowiak P, Drag M, Materna V, Suchocki S, Grzywa R, Spaczyński M, et al: Expression of aminopeptidase N/CD13 in human ovarian cancers. Int J Gynecol Cancer 2006;16:1783–88. 10.1111/j.1525-1438.2006.00657.x 17009972

[13] Kessler T, Schwöppe C, Liersch R, Schliemann C, Hintelmann H, Bieker R, et al: Generation of fusion proteins for selective occlusion of tumor vessels. Current Drug Discovery Technologies 2008;5:1–8. 10.2174/157016308783769487 18537561

[14] Bieker R, Kessler T, Schwöppe C, Padró T, Persigehl T, Bremer C, et al: Infarction of tumor vessels by NGR-peptide-directed targeting of tissue factor: experimental results and first-in man experience. Blood 2009;113:5019–27. 10.1182/blood-2008-04-150318 19179306

[15] Schwöppe C, Kessler T, Persigehl T, Liersch R, Hintelmann H, Dreischalück J, et al: Tissue-factor fusion proteins induce occlusion of tumor vessels. Thrombosis Research 2010;125 Suppl 2:S143–50. 20433995 10.1016/S0049-3848(10)70033-5

[16] Schwöppe C, Zerbst C, Fröhlich M, Schliemann C, Kessler T, Liersch R, et al: Anticancer therapy by tumor vessel infarction with polyethylene glycol conjugated retargeted tissue factor. J Med Chem 2013;56:2337–47. 10.1021/jm301669z 23496322

[17] Persigehl T, Ring J, Bremer C, Heindel W, Holtmeier R, Stypmann J, et al: Non-invasive monitoring of tumor-vessel infarction by retargeted truncated tissue factor tTF-NGR using multi-modal imaging. Angiogenesis 2014;17:235–46. 10.1007/s10456-013-9391-4 24136410

[18] Stucke-Ring J, Ronnacker J, Brand C, Höltke C, Schliemann C, Kessler T, et al: Combinatorial effects of doxorubicin and retargeted tissue factor by intratumoral entrapment of doxorubicin and proapoptotic increase of tumor vascular infarction. Oncotarget 2016;7:82458–472. 10.18632/oncotarget.12559 27738341 PMC5347705

[19] Ruf W, Rehemtulla A, Morrissey JH, Edgington TS: Phospholipid-independent and -dependent interactions required for tissue factor receptor and cofactor function. J Biol Chem 1991;266:2158–66, (erratum J Biol Chem 1991;266: 16256). 1989976

[20] Dachary-Prigent J, Toti F, Satta N, Pasquet J-M, Uzan A, Freyssinet J-M: Physiopathological significance of catalytic phospholipids in the generation of thrombin. Semin Thromb Hemost 1996;22:157–64. 10.1055/s-2007-999004 8807713

[21] Bach R, Gentry R, Nemerson Y: Factor VII binding to tissue factor in reconstituted phospholipid vesicles: induction of cooperativity by phosphatidylserine. Biochemistry 1986;25:4007–20. 10.1021/bi00362a005 3527261

[22] Bombeli T, Karsan A, Tait JF, Harlan JM: Apoptotic vascular endothelial cells become procoagulant. Blood 1997;89:2429–42. 9116287

[23] Blankenberg FG, Katsikis PD, Tait JF, Davis RE, Naumovski L, Ohtsuki K, et al: In vivo detection and imaging of phosphatidylserine expression during programmed cell death. Proc Natl Acad Sci USA 1998;95:6349–54. 10.1073/pnas.95.11.6349 9600968 PMC27696

[24] Hu S, Kiesewetter DO, Zhu L, Guo N, Gao H, Liu G, et al: Longitudinal PET imaging of doxorubicin-induced cell death with 18F-annexin V. Mol Imaging Biol 2012;14:762–70. 10.1007/s11307-012-0551-5 22392643 PMC3387344

[25] Swystun LL, Shin LYY, Beaudin S, Liaw PC: Chemotherapeutic agents doxorubicin and epirubicin induce procoagulant phenotype on endothelial cells and blood monocytes. J Thromb Haemost 2009;7:619–26. 10.1111/j.1538-7836.2009.03300.x 19187077

[26] Lysov Z, Swyston LL, Kuruvilla S, Arnold A, Liaw PC: Lung cancer chemotherapy agents increase procoagulant activity via protein disulfide isomerase-dependent tissue factor decryption. Blood Coagul Fibrinolysis 2015;26:36–45. 10.1097/MBC.0000000000000145 24911456

[27] Brand C, Dencks S, Schmitz G, Mühlmeister M, Stypmann J, Ross R, et al: Low-energy ultrasound treatment improves regional tumor vessel infarction by retargeted tissue factor. J Ultrasound Med 2015;34:1227–36. 10.7863/ultra.34.7.1227 26112625

[28] Carmeliet P, Jain RK: Molecular mechanisms and clinical applications of angiogenesis. Nature 2011;473:298–307. 10.1038/nature10144 21593862 PMC4049445

[29] He J, Luster TA, Thorpe PE: Radiation-enhanced vascular targeting of human lung cancers in mice with a monoclonal antibody that binds anionic phospholipids. Clin Cancer Res 2007;13:5211–18. 10.1158/1078-0432.CCR-07-0793 17785577

[30] Berdel WE, Schick P, Sedlmeier H, Fink U, Rastetter J, Messerschmidt O: Experimental chemotherapy of radiation injury with synthetic lysophospholipid analogs in mice. Radiation Res 1983;94:166–70. 6344130

[31] Bhagwat SV, Lahdenranta J, Giordano R, Arap W, Pasqualini R, Shapiro LH: CD13/APN is activated by angiogenic signals and is essential for capillary tube formation. Blood 2001;97:652–59. 10.1182/blood.v97.3.652 11157481 PMC4470622

[32] Faintuch BL, Oliveira EA, Targino RC, Moro AM: Radiolabeled NGR phage display peptide sequence for tumor targeting. Appl Radiation Isotopes 2014;86:41–45. 10.1016/j.apradiso.2013.12.035 24480451

[33] Mate G, Kertesz I, Enyedi KN, Mezö G, Angyal J, Vasas N, et al: In vivo imaging of aminopeptidase N (CD13) receptors in experimental renal tumors using the novel radiotracer 68Ga-NOTA-c(NGR). Eur J Pharm Sci 2015;69:61–71. 10.1016/j.ejps.2015.01.002 25592229

[34] Oostendorp M, Douma K, Hackeng TM, Dirksen A, Post MJ, van Zandvoort MAMJ, et al: Quantitative molecular magnetic resonance imaging of tumor angiogenesis using cNGR-labeled paramagnetic quantum dots. Cancer Res 2008;68:7676–83. 10.1158/0008-5472.CAN-08-0689 18794157

[35] Corti A, Curnis F, Rossoni G, Marcucci F, Gregorc V: Peptide-mediated targeting of cytokines to tumor vasculature: the NGR-hTNF example. BioDrugs 2013;27:591–603. 10.1007/s40259-013-0048-z 23743670 PMC3832761

[36] Gregorc V, Zucali PA, Santoro A, Ceresoli GL, Citterio G, de Pas TM, et al: Phase II study of asparagine-glycine-arginine-human tumor necrosis factor alpha, a selective vascular targeting agent, in previously treated patients with malignant pleural mesothelioma. J Clin Oncol 2010;28:2604–11. 10.1200/JCO.2009.27.3649 20406925

[37] Ferreri AJM, Calimeri T, Conte GM, Cattaneo D, Fallanca F, Ponzoni M, et al R-CHOP preceded by blood-brain barrier permeabilization with engeneered tumor necrosis factor-alpha in primary CNS lymphoma. Blood 2019 5 22; pii: blood.2019000633 10.1182/blood.2019000633 31118164

[38] Bauvois B, Dauzonne D: Aminopeptidase-N/CD13 (EC 3.4.11.2) inhibitors: chemistry, biological evaluations, and therapeutic prospects. Medicinal Res Rev 2006;26:88–130. 10.1002/med.20044 PMC7168514 16216010

[39] Ichinose Y, Genka K, Koike T, Kato H, Watanabe Y, Mori T, et al: Randomized double-blind placebo controlled trial of bestatin in patients with resected stage I squamous-cell cancer. J Natl Cancer Inst 2003;95:605–10. 10.1093/jnci/95.8.605 12697853

